# Risk factors for respiratory work disability in a cohort of pulp mill workers exposed to irritant gases

**DOI:** 10.1186/1471-2458-11-689

**Published:** 2011-09-06

**Authors:** Nicola Murgia, Kjell Torén, Jeong-Lim Kim, Eva Andersson

**Affiliations:** 1 Occupational and Environmental Medicine, Sahlgrenska University Hospital, Gothenburg, Sweden; 2 Section of Occupational Medicine, Respiratory Diseases and Toxicology, University of Perugia, Perugia, Italy

## Abstract

**Background:**

The association between chronic respiratory diseases and work disability has been demonstrated a number of times over the past 20 years, but still little is known about work disability in occupational cohorts of workers exposed to respiratory irritants. This study investigated job or task changes due to respiratory problems as an indicator of work disability in pulp mill workers occupationally exposed to irritants.

**Methods:**

Data about respiratory symptoms and disease diagnoses, socio-demographic variables, occupational exposures, gassing episodes, and reported work changes due to respiratory problems were collected using a questionnaire answered by 3226 pulp mill workers. Information about work history and departments was obtained from personnel files. Incidence and hazard ratios for respiratory work disability were calculated with 95% confidence intervals (CI).

**Results:**

The incidence of respiratory work disability among these pulp mill workers was 1.6/1000 person-years. The hazard ratios for respiratory work disability were increased for workers reporting gassings (HR 5.3, 95% CI 2.7-10.5) and for those reporting physician-diagnosed asthma, chronic bronchitis, and chronic rhinitis, when analyzed in the same model.

**Conclusions:**

This cohort study of pulp mill workers found that irritant peak exposure during gassing episodes was a strong predictor of changing work due to respiratory problems, even after adjustment for asthma, chronic bronchitis, and chronic rhinitis.

## Background

There is increasing scientific evidence indicating that asthma is an important predictor of work disability [1]. This evidence mainly comes from large general population-based studies such as the European Community Respiratory Health Survey (ECRHS), in which the prevalence of respiratory work disability, defined as changing or leaving work due to affected breathing, was found to range from 1.6% to 8.1% in various countries [2]. In the follow-up of the ECRHS population, the incidence of respiratory work disability was 1.2/1000 person-years in the total population and 5.7/1000 person-years among participants with asthma [3]. The corresponding cumulative prevalences were 1.1% and 4.9%, respectively. Exposure to air pollution at the workplace was an important predictor of respiratory work disability, both in the cross-sectional analysis [2] and in the longitudinal analysis [3]. In the longitudinal study, the exposure was divided into three groups; organic dust, mineral dust, and irritant gases and fumes, and the highest risk for respiratory work disability was observed among those exposed to mineral dust. However, there was a moderate but not significant increased risk among those exposed to irritant gases and fumes. Hence, the importance of exposure to irritant gases and fumes remains obscure and needs to be further evaluated.

We have previously demonstrated that occupational exposure to irritant gases, especially peak exposure, is associated with increased incidence of adult-onset asthma in pulp mill workers [4,5]. We now hypothesize that peak exposure to irritants such as sulphur dioxide and chlorine dioxide increases the risk of respiratory work disability.

The aim of the present study was thus to investigate the incidence of respiratory work disability among pulp and paper workers, defined as changing work due to respiratory problems, and to evaluate the potential linkage with occupational exposure to irritants as well as respiratory diseases and other predisposing factors.

## Methods

### Study design and subjects

In this study, we examined a cohort of Swedish pulp and paper mill workers from four sulphite mills and one sulphate (Kraft) mill established to study different effects of irritants. The main occupational exposures in pulping are wood dust, terpenes, and bleachery chemicals such as chlorine and chlorine dioxide. Sulphite pulping involves exposure to sulphur dioxide, especially in the digester, and sulphate pulping also involves exposure to hydrogen sulphide and other reduced sulphur compounds [6].

The cohort was established by identifying workers from the personnel files in each mill. The initial cohort comprised 14175 subjects employed in the mills for six months or more at any time between 1940 and 2000. A questionnaire was mailed to all members of this cohort who had been employed at any time between 1 January 1970 and 1 July 2000, who were aged under 80 years, and who were alive on 1 July 2000 (n = 7389). Most of the items on this questionnaire had been validated and used in previous studies [7,8].

The response rate was 52.7% (n = 3894). Currently employed were more prone to respond (62.2%) compared to not currently employed (44.1%). Workers employed more than 10 years and workers aged 50-64 years, answered more frequently than the others. Of the responders 9% answered to the last reminder, a shorter version of the questionnaire survey containing the key questions about respiratory diseases and occurrence of gassing, but not on work change. In addition to the questionnaire data, information for all subjects was extracted from personnel files regarding time period of employment, job title, and mill department. The final population (n = 3226) for this study comprised subjects identified in the personnel files with complete data regarding both employment periods and respiratory work disability.

The local ethics committee at University of Gothenburg reviewed and approved the study protocol.

### Definitions

Respiratory work disability was defined as an affirmative answer to the question "Have you ever had to change your job or task due to respiratory problems?"; the reported year in which this change occurred was also recorded [9].

Asthma was defined as self-reported physician-diagnosed asthma [7].

Childhood asthma was defined asthma with reported debut before 16 years of age.

Chronic bronchitis was defined as self-reported cough and phlegm for at least three months for two consecutive years or longer [10].

Chronic rhinitis was defined as rhinitis after the age of 15 that lasted more than a month [11].

Allergic status was defined as an affirmative answer to questions about allergy in childhood and/or ever having had hay fever.

Participants were divided according to smoking status, into current smokers, ex-smokers, and never-smokers. Ever-smokers were defined as workers who had ever smoked daily for at least one year. The years of starting and stopping smoking were used to create a time-dependent variable for smoking habits. Exposure to environmental tobacco smoke (ETS) in the workplace was defined as self-reported exposure to ETS several times a week over the years worked at the pulp mill.

Exposure to irritant gassings was defined either as "gassings" or as having worked in those departments with a high probability of exposure to irritant gases, that is, the digester and bleachery departments. Gassing was defined as a positive answer to at least one of the three sections of the question [8]: "Have you been exposed to i) sulphur dioxide, ii) chlorine/chlorine dioxide, or iii) any other irritant substances at work, resulting in coughing, breathlessness, wheezing, or chest pain?". When analyzing gassings, the unexposed workers were those who did not report any gassing episode at work. Workers in the digester and bleachery departments were compared with workers in the other departments, such as paper production departments.

### Statistical methods

We only considered the periods when the subjects were employed in the pulp mills. The observation period was retrospective: 1970-2000. Person-years were calculated from start of employment until end of employment period (according to data from personnel files) or until the year of first occurrence of respiratory work disability. Incidence of respiratory work disability was calculated for the various disease categories; the incidence rate ratio (IRR) and its 95% test-based confidence intervals (CI) for respiratory work disability were computed by comparing workers reporting gassings (irritant peak exposures) with unexposed workers [12].

In the final analysis, the hazard ratios (HR) and 95% CI for respiratory work disability were modelled using Cox proportional hazards regression models. The outcome event was respiratory work disability, and time until event was determined by reported date of first occurrence of respiratory work disability. The exposure was analyzed either as gassings or as work in exposed departments adjusting for asthma, chronic bronchitis, chronic rhinitis, and passive smoking at work. Time-dependent smoking habits, time-dependent age more than 50 years, and allergic status were not included in the final model, and exclusion from the model had minimal impact on coefficients for the exposures of interest.

Sex was not included in the model, as only one woman reported respiratory work disability. All statistical analysis was performed using the statistical software packages SAS version 9.2 (SAS Institute, Cary, NC, USA).

## Results

The characteristics of the study population and prevalence of respiratory work disability are shown in Table 1. The person-years of the entire cohort totalled 47734. The incidence of respiratory work disability was 1.6/1000 person-years (95% CI 1.3-2.0) for all pulp mill workers, 2.9/1000 person-years (95% CI 2.3-3.7, n = 66) for workers who reported gassing, and 0.4/1000 person-years (95% CI 0.2-0.7, n = 10) for workers who did not report gassing. The incidence rate ratio was 7.3 (95% CI 4.1-12.9).

**Table 1:** Characteristics of a cohort of pulp mill workers and prevalence of respiratory work disability during employment time in pulp and paper mills

Subject characteristics	Total	Respiratory work disability	
	N	%	N
Pulp mill workers	3226	2.4	76
Men	2733	2.7	75
Women	493	0.2	1
Allergic status	583	3.8	22
Never-smokers	1678	2.3	38
Ex-smokers	1129	2.6	29
Current smokers	419	2.1	9
ETS exposure at work in pulp industry	1654	3.2	53
Work in digester department	389	5.9	23
Work in bleachery department	200	4.5	9
Reported gassings	1283	5.1	66
Asthma (including childhood asthma)	235	14.9	35
Childhood asthma	63	4.8	3
Chronic bronchitis	205	11.2	23
Chronic rhinitis	524	6.1	32

Among the 774 subjects with asthma, chronic bronchitis, and/or chronic rhinitis, 57 reported change of work. The incidence of respiratory work disability was 10.2/1000 person-years (95% CI 8.6-16.5, n = 35) among those with asthma, 6.7/1000 person-years (95% CI 4.5-10.1, n = 23) among those with chronic bronchitis, and 4.7/1000 person-years (95% CI 3.4-6.8, n = 32) among those with chronic rhinitis; it should be noted that there was some overlap in these conditions. About half of the subjects with any of these respiratory diseases developed their disease during employment in pulp mills. Of the participants with chronic bronchitis, 62% were ever-smokers.

Occupational exposure defined as reported gassings was associated with an increased risk of respiratory work disability (HR 5.3, 95% CI 2.7-10.5; see Table 2). Asthma was also a strong risk factor for respiratory work disability (HR 6.7, 95% CI 4.1-11.0) but excluding asthma from the model did not substantially change the estimate for gassings (from HR 5.3 to 5.8). Smoking, allergic status, and age did not influence the risk of respiratory work disability. Exposure to gassings was a risk factor for respiratory work disability both among asthmatics and workers without asthma, chronic bronchitis and/or chronic rhinitis (HR 5.4, 95% CI 1.9-15 and HR 8.3, 95% CI 2.4-29 respectively). The risk of respiratory work disability during pulp mill work was similar for workers with pre-employment asthma and asthma with onset during employment, when adjusted for gassings (data not shown).

**Table 2:** Hazard ratios (HR) with 95% confidence intervals (CI) for respiratory work disability (i.e. changing work due to respiratory symptoms) in a cohort of pulp mill workers exposed to irritant gases (gassings).

**Variable **^1^	HR	95% CI
Exposure (gassings) ^2^	5.3	2.7-10.5
Asthma^2^	6.7	4.1-11.0
Chronic bronchitis^2^	1.8	1.1-3.1
Chronic rhinitis^2^	1.8	1.1-3.0
ETS exposure at work in pulp industry^2^	1.5	0.9-2.5

1; Mutual adjustments for the variables expressed with HR

2; Yes = 1, No = 0

When using work department to measure exposure, working in the digester department was associated with an increased risk of respiratory work disability (HR 2.1, 95% CI 1.2-3.4), but working in the bleachery department was not a strong risk factor (HR 1.5, 95% CI 0.8-3.1). However, in these models asthma remained as a strong risk factor for respiratory work disability (data not shown).

## Discussion

Our main finding in this cohort of pulp and paper mill workers was the association between irritant peak exposures and respiratory work disability, defined as change of work due to affected breathing. In addition, asthma, but also chronic bronchitis and chronic rhinitis, played an important role in causing work change.

In this cohort, the incidence of respiratory work disability was 10.2/1000 person-years among subjects with asthma. This estimate is almost twice that found in asthmatics from the general population in the ECRHS (5.7/1000 person-years). The incidence rate among all workers in this study was 1.6/1000 person-years, which is quite similar to the estimate in the ECRHS general population sample (1.2/1000 person-years) [3]. This could be explained by the fact that irritant exposure was fairly common; 40% of our study population reported gassings. The longitudinal analysis of the ECRHS study revealed an indication (not significant) of an exposure-response relationship between exposure to gases and fumes and respiratory work disability, both among asthmatics and in the general population. This indicates that higher exposure may increase the risk of respiratory work disability.

Most previous studies in this area were performed using population-based surveys. To our knowledge, this is one of only a few studies of respiratory work disability to examine an occupational cohort, pulp and paper mill workers in this case. Pulp production in the paper industry involves known respiratory irritants such as sulphur dioxide and chlorine dioxide, especially in the digester and bleachery departments, where we found a higher prevalence of work change. The prevalence of gassing in our study population (40%) was similar to that found in previous studies (45-60%); gassing occurs fairly frequently in the pulp industry, but is often not severe enough to require health care (e.g., physician intervention or hospitalization) and sick leave from work [10,13,14], so it does not necessarily lead to change of work. Some authors have addressed the issue of increased asthma risk in pulp and paper industry workers, especially in those who have experienced gassing [13,14]. In this study asthma could be both a confounder (that is related to gassings and independently related to disability without being involved in the causal pathway) and an intermediate between the exposure and the outcome of disability. If asthma mainly acted as an intermediate the risk of disability associated with gassings should differ with and without asthma, but that has not occurred in our analyses. Workers with pre-employment asthma, as well as workers without respiratory diseases, were also at risk for disability. We found gassing to be a strong and independent predictor of changing work due to respiratory problems.

The present study did not suggest a strong effect of smoking habits on work change. This "healthy smoker effect" seems to be amplified in those with respiratory diseases, especially if the onset is in childhood or adolescence, in which case the subject is usually less likely to start smoking [15]. Workplace ETS seems to be a weak risk factor for changing work.

It was not possible to study the influence of sex in this cohort, since only one woman reported changing work due to respiratory problems.

The definition of work change as an indicator of work disability was formulated on the basis of previous surveys, and was preferred to a self-reported work ability scale [3,16]. In any case, the key questions about exposure may be double-biased by respiratory symptoms and the presence of work change due to respiratory symptoms. Prompted questions, such as those in the present questionnaire, seem less prone to recall bias than open-ended questions, and questions about irritant peak exposure have been used in other epidemiological studies [13,14,17].

The response rate was not high and could have influenced the result. The shorter questionnaire version was used for a random non-responders interview (n = 254) in the cohort. Items on work change were not included, but the risk of asthma related to gassings among non-responders was similar to responders despite differences in age and employment [5]. Also the risk estimate and CI are so clearly significant that less risk among non-responders would not change the conclusion.

## Conclusions

This is the first study of the impact of workplace exposure on work change in an occupational cohort of subjects exposed to irritants and likely to experience gassing episodes. Our findings suggest that, in the pulp industry, gassing could affect not only airway hyper-responsiveness but also work ability. Moreover, asthma and other respiratory diseases rendered workers more likely to change work due to respiratory problems. Therefore, more attention should be paid to preventing accidents that result in even transient occupational exposure to irritants, especially for workers already affected by respiratory disorders.

## List of abbreviations

CI: confidence interval; ECRHS: European Community Respiratory Health Survey; ETS: environmental tobacco smoke; HR: hazard ratio; IRR: incidence rate ratio

## Competing interests

The authors declare that they have no competing interests.

## Authors' contributions

KT and EA designed the study; EA and KT were responsible for data collection; EA managed the data; NM, EA, and J-LK analyzed the data. All authors participated in the interpretation and final drafting of the manuscript. All authors read and approved the final manuscript.

## Pre-publication history

The pre-publication history for this paper can be accessed here:

http://www.biomedcentral.com/1471-2458/11/689/prepub
